# Supplementary material for: Development of a New Nomogram Including Neutrophil-to-Lymphocyte Ratio to Predict Survival in Patients with Hepatocellular Carcinoma Undergoing Transarterial Chemoembolization
Source: Cancers (Basel). 2019 Apr 10;11(4):509. doi: 10.3390/cancers11040509 (PMC6520830; doi:10.3390/cancers11040509)
Supplement: Supplementary file 1 [file cancers-11-00509-s001.pdf]

Supplementary Material: Development of a New Nomogram Including Neutrophil-to-Lymphocyte Ratio to Predict Survival in Patients with Hepatocellular Carcinoma Undergoing Transarterial Chemoembolization

Young Eun Chon, Hana Park, Hye Kyung Hyun, Yeonjung Ha, Mi Na Kim, Beom Kyung Kim, Joo Ho Lee, Seung Up Kim, Do Young Kim, Sang Hoon Ahn, Seong Gyu Hwang, Kwang-Hyub Han, Kyu Sung Rim and Jun Yong Park

Table S1. Univariate Cox regression analysis of potential predictors in the derivation set (n = 921).

**Variables**	**Univariate p-value**
Age (years)	0.811
Male gender	0.913
Etiologies (HBV vs. HCV or alcohol or others)	0.361
AFP (ng/mL)	<0.001
AFP (≥200 vs. <200 ng/mL)	<0.001
∆ AFP	0.354
Tumor size (cm)	<0.001
Tumor size (>3cm vs. ≤3cm)	<0.001
Tumor number (>3 vs. ≤3)	<0.001
Vascular invasion	<0.001
BCLC stage (C or B vs. A)	<0.001
Performance status (1–2 vs. 0)	0.161
Child-Pugh score (6 or 7–8 or 9 vs. 5)	<0.001
Child-Pugh class (B vs. A)	<0.001
∆ Child-Pugh score	0.235
Treatment response (SD+PD vs. CR + PR)	<0.001
AST (IU/L)	0.437
∆ AST	0.320
ALT (IU/L)	0.680
∆ ALT	0.204
Serum albumin (g/dL)	<0.001
Total bilirubin (g/dL)	0.011
Prothrombin time (INR)	0.173
Platelet (10^9/L)	0.098
NLR ratio	<0.001
NLR ratio (≥5 vs. <5)	<0.001
∆ NLR ratio	0.786

HBV, hepatitis B; HCV, hepatitis C; AFP, alpha-feto protein; BCLC, the Barcelona clinic liver cancer, AST, aspartate aminotransferase, ALT,alanine transaminase, NLR, Neutrophil-to-lymphocyte ratio. ∆ stands for value difference between baseline and 1 month after TACE.

Table S2. Predictive performances of SNAVCORN score vs. SNAVCN or SNAVCOR score to predict overall survival.

	Time-dependent AUROC (95% CI)			*p*-value	*p*-value
Year	SNAVCORN	SNAVCN	SNAVCOR	SNAVCORN vs. SNAVCN	SNAVCORN vs. SNAVCOR
Year1	0.812 (0.769–0.856)	0.795 (0.750–0.840)	0.797 (0.762–0.832)	<0.001	<0.001
Year3	0.734 (0.396–0.770)	0.723 (0.696–0.769)	0.705 (0.693–0.746)	0.108	<0.001
Year5	0.700 (0.663–0.737)	0.679 (0.661–0.736)	0.665 (0.619–0.733)	0.035	0.019

AUROC, area under receiver-operating characteristic curve; CI, confidence interval. SNAVCORN score features baseline tumor Size (≥5 cm), tumor Number (≥4), AFP level (≥400 ng/mL), presence of vascular invasion, Child-Pugh score, the absence of Objective Response after TACE, and NLR (≥5). SNAVCN score features baseline tumor Size (≥5 cm), tumor Number (≥4), AFP level (≥400 ng/mL), presence of vascular invasion, Child-Pugh score, and NLR (≥5). SNAVCOR score features baseline tumor Size (≥5 cm), tumor Number (≥4), AFP level (≥400 ng/mL), presence of vascular invasion, Child-Pugh score, and the absence of Objective Response after TACE.

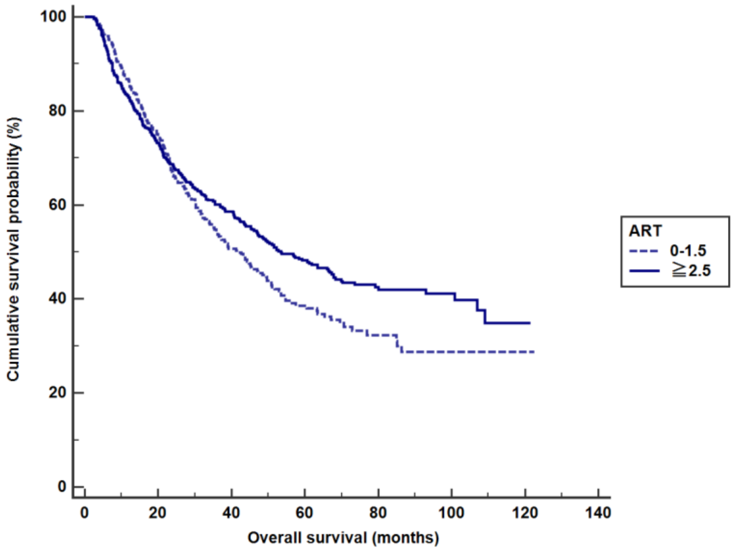

(a)

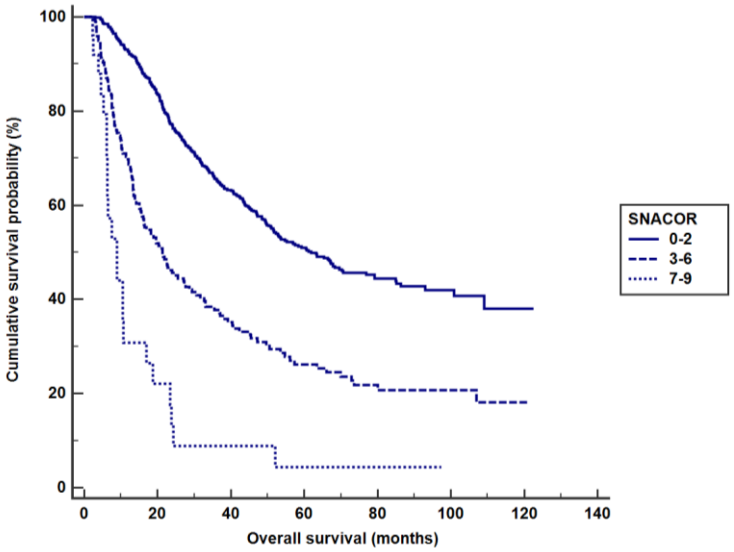

(b)

Figure S1. *Cont.*

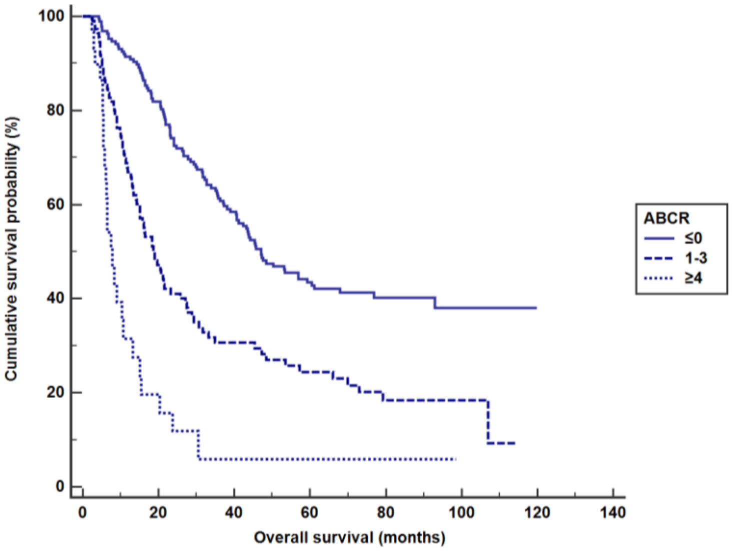

(c)

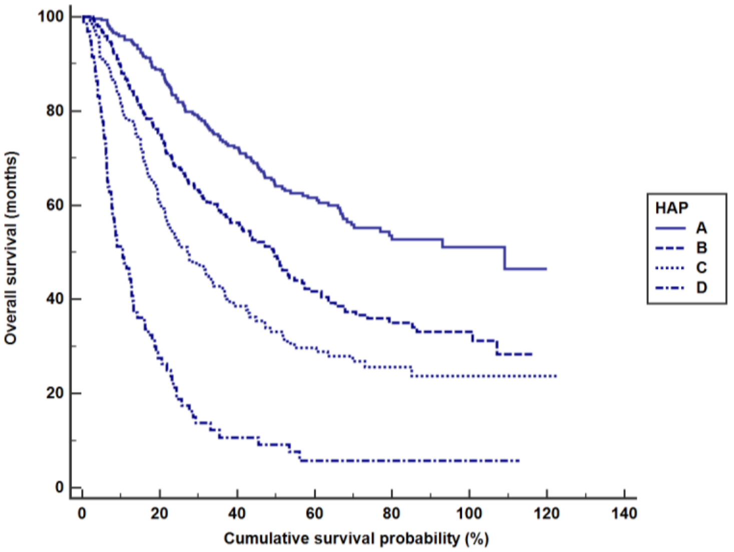

(d)

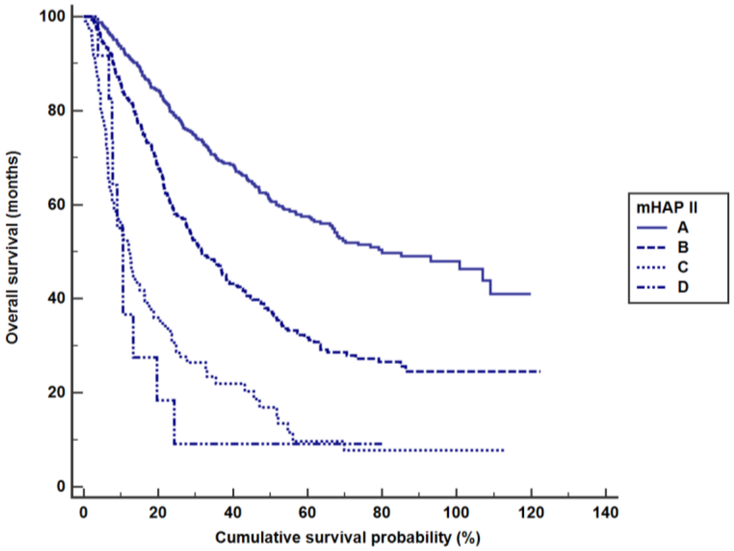

(e)

Figure S1. Comparison of the Kaplan-Meier overall survival curves for risk prediction models in patients with hepatoceullar carcinoma undergoing transarterial chemoembolization. (**a**) ART score (**b**) SNACOR model (**c**) ABCR score (**d**) HAP Score (**e**) mHAP II score.
